# A Pre-Post Study of Individualized Programs Using the Occupational Therapy Intervention Process Model in a Psychiatric Hospital in Japan

**DOI:** 10.7759/cureus.86918

**Published:** 2025-06-28

**Authors:** Yusuke Imamoto, Yasushi Orita, Hiromi Yoshikawa, Ryotaro Tsukue, Kenichi Tokumitsu, Misaki Nagai, Natsuki Yorozuya, Koichiro Fujimaki

**Affiliations:** 1 Department of Occupational Therapy, Faculty of Health and Welfare, Prefectural University of Hiroshima, Mihara, JPN; 2 Department of Psychiatry, Senogawa Hospital, Hiroshima, JPN

**Keywords:** addiction treatment, assessment of motor and process skills, canadian occupational performance measure, evaluation of social interaction, individualized rehabilitation, occupational therapy intervention process model, psychiatric hospital, psychiatric occupational therapy, psychiatric rehabilitation, schizophrenia and other psychotic disorders

## Abstract

Background: Recent psychiatric treatment trends emphasize supporting individuals to live autonomously within the community, irrespective of the severity of mental illness. This study investigates the effects of individualized programs based on the Occupational Therapy Intervention Process Model (OTIPM) on occupational performance and social interaction skills among psychiatric inpatients.

Methodology: This pre-post study included 90 clients (schizophrenia: 46, addiction: 15, mood disorders: 11, intellectual disability: 7, others: 11) hospitalized in a psychiatric facility. Participants underwent a maximum of 10 individualized sessions using OTIPM. Outcomes assessed were the Canadian Occupational Performance Measure (COPM), Assessment of Motor and Process Skills (AMPS), and Evaluation of Social Interaction (ESI). Statistical analyses included paired t-tests and Wilcoxon signed-rank tests.

Results: Significant improvements were observed in COPM performance (*p* < 0.001, Cohen's d = 0.79), COPM satisfaction (*p* < 0.001, Cohen's d = 0.97), AMPS motor skills (*p *< 0.001, Cohen's d = 0.61), AMPS process skills (*p* < 0.001, Cohen's d = 0.59), and ESI scores (*p* < 0.001, Cohen's d = 0.72). Improvements were consistent regardless of the frequency of sessions or the length of hospitalization.

Conclusions: Individualized interventions based on OTIPM may effectively enhance occupational performance and social interaction skills, providing valuable support for community reintegration among psychiatric inpatients.

## Introduction

In recent years, efforts have been made to build a comprehensive community care system for people with mental disorders [1], and psychiatric treatment has shifted from being based on long-term hospitalization in psychiatric hospitals to providing support so that everyone, regardless of the degree of mental disorder, can live their own life as a member of the community with peace of mind.

While the environment surrounding psychiatric treatment is changing, group-based occupational therapy programs, which are considered to be cost-effective, continue to be widely implemented. However, programs specifically designed for the acute phase or to facilitate early discharge are still limited in number. On the other hand, few acute-phase programs or programs to promote discharge have been implemented [2]. One possible reason for the limited implementation of early discharge programs is that, under Japan's national health insurance system, the provision of group-based programs is considered the standard approach. The insurance system was established in 1974 and partially revised in 2006, but there have been no major changes since then [3].

In order to support people suffering from psychiatric disorders to live their own life with peace of mind as a member of the community, it is not sufficient to implement group programs alone, and an individual approach in line with the needs of each person is important [4]. The implementation of an individual program that utilizes the expertise of occupational therapists (OT) supports clients to live their own lives with peace of mind as members of the community. In addition, because of examining the factors related to the outcome of patients with schizophrenia one year after discharge, it has been reported that goal-oriented individual occupational therapy during hospitalization and good outpatient attendance and medication compliance after discharge are effective in preventing re-hospitalization of patients with schizophrenia [5].

The Occupational Therapy Intervention Process Model (OTIPM), recommended by Fisher as a true top-down approach, is a method to support a person's unique life [6]. OTIPM is a client-centered, occupation-based approach characterized by the client’s actual engagement in meaningful occupations [7]. One of its notable features is the incorporation of direct observation of occupational performance into the evaluation process. By observing the client performing real tasks, a shared understanding between the OT and the client can be established, which in turn facilitates collaborative goal setting and intervention. OTIPM consists of three phases: evaluation and goal setting, intervention, and re-evaluation. While engaging in actual occupations, therapists and clients collaborate flexibly, moving back and forth between these phases as needed. The intervention phase includes four models: the compensatory model, the education model, the acquisitional model, and the restorative model. Therapists implement interventions by combining these models appropriately based on the client's needs.

In contrast, group occupational therapy programs commonly implemented in Japanese psychiatric settings typically involve approximately 20 clients participating in light exercise, creative activities, or music sessions. The content of these programs is usually determined by the OT in charge, and clients select from the available options and join the sessions. As a result, these programs do not necessarily provide direct intervention targeting occupations that are meaningful or important to the individual client.

Given these characteristics, the OTIPM may allow for more effective intervention in facilitating client discharge by directly addressing the challenges and difficulties encountered in their daily lives.

Imamoto et al. practiced an OTIPM-based individual occupational therapy program with 10 schizophrenic patients in an open ward of a psychiatric hospital and analyzed the treatment effects [8]. The study suggested that the occupation-based individual program showed significant improvements in the comparison of effects on the Evaluation of Social Interaction (ESI) and Frontal Assessment Battery scores, compared to the group occupational therapy program. Furthermore, Imamoto et al. implemented an OTIPM-based individual program for alcohol- and drug-dependent patients admitted to a psychiatric hospital, and compared the results before and after the program, and found significant improvements in performance on the Canadian Occupational Performance Measure (COPM), satisfaction, and social interaction skills [9].

Despite these previous studies, a scoping review conducted by Sasaki & Tanimura on early occupational therapy for inpatients with psychiatric disorders in Japan revealed several issues for future research [10]. These included unclear objectives in many reports of group programs and a lack of studies reporting occupation-related outcomes [10]. Based on these findings, we believe that practice-based reports and studies focusing on occupational outcomes for clients are essential for the further development of psychiatric occupational therapy.

In the present study, we aimed to clarify how the implementation of an individual program based on the OTIPM affects occupational performance and social interaction skills, not only in clients with specific disorders such as schizophrenia or addiction, but also in those with a variety of psychiatric conditions. Additionally, we sought to examine whether the effects differ depending on the frequency of program implementation and the duration of hospitalization prior to the start of the program.

## Materials and methods

Participants

This study employed a single-group pre-post design to examine changes in outcomes before and after the implementation of an individualized program based on the OTIPM. Participants were recruited using a consecutive sampling method. The study was conducted between January 2017 and March 2021, during which 117 clients participated in the individualized program. All clients began the program while hospitalized and remained hospitalized until the program was completed.

The inclusion criterion was participation in the program and completion of the final evaluation during the study period. The exclusion criterion was clients who had expressed willingness to participate and completed the initial evaluation but were unable to proceed with the program. As a result, 27 clients were excluded, and a total of 90 clients were included in the final analysis.

Participation in the program was determined by individual interviews with the OT, requests from the clients themselves, instructions from the attending physician, and requests from other professions, such as the nurse in charge. The program participants were informed orally using an explanatory document, and their consent to participate in the program was obtained in writing.

This study was approved by the Ethics Committee of Senogawa Medical Corporation (Hiroshima, Japan) on June 16, 2022. The approval number is R04-11.

Intervention methods

In our individualized program, we shared that all staff members were aware of the OTIPM process when practicing and always kept the focus on the occupation of the client. Table 1 shows the differences between the group occupational therapy programs commonly implemented in Japan and the individualized programs based on the OTIPM.

**Table 1:** Differences between group and individual programs OTIPM, Occupational Therapy Intervention Process Model; OT, occupational therapist; CL, client

Category	Group programme	Individual programme
Intervention method	Group exercise, creative activities, etc.	OTIPM
Importance of the tasks addressed	Unknown importance to CL	Important to CL
Programme content	Basically decided by OT	CL and OT decide
Reflection	Irregular	Conducted every time
Period of implementation	No upper limit	Maximum 10 sessions

Interventions and evaluations were carried out by OTs from the hospital, including the author. The clinical experience of the staff involved in the individualized programs based on OTIPM is summarized in Table 2.

**Table 2:** Clinical experience of intervention staff

Years of clinical experience	Number of staff
More than 10 years	2
8 years	1
7 years	2
6 years	1
4 years	2
3 years	3
2 years	1
1 year	1

Training sessions on the OTIPM and COPM are held annually at the hospital. As the content is generally consistent each year, more experienced staff tend to participate more frequently. Case debriefing sessions are conducted regularly, and the author provides feedback as needed during interventions to ensure a consistent level of service. First-year staff members do not conduct the interventions alone, but do so with the support of senior staff.

During the study period, all clients were provided with both the individualized program and the regular occupational therapy program. In the regular occupational therapy program, interventions were sometimes conducted by occupational therapists other than those in charge of the individualized program.

The intervention process in this practice consisted of three stages: occupation assessment and occupation goal setting, occupation-focused intervention, and occupation reassessment. The program was set at 60 minutes per session, and the maximum number of sessions was 10. These settings were determined by taking into account what occupational therapy staff could realistically work on while carrying out their regular occupational therapy program. As the length of stay in an acute ward is approximately three months, this is a number of sessions that can be completed without difficulty if the program is delivered once a week. In some cases, programs were run at a high frequency over a short period of time, or clients felt that they had solved their operational problems and wished to finish the program before reaching 10 sessions.

Examples of specific program activities included cooking tasks, computer operation practice, creative activities, and work experience. For example, in cooking activities, the client and therapist work together to decide on a menu and purchase ingredients. Through the actual cooking process, the client is encouraged to reflect on their experiences, share insights gained, and consider ways to improve their performance.

Outcome

In our previous study, changes in occupational performance and social interaction skills were observed [9]. Therefore, COPM, the Assessment of Motor and Process Skills (AMPS), and ESI were selected as outcome measures for this study [11-13]. The evaluations were conducted by OTs who were part of the research team. The assessments were conducted twice: once before and once after the implementation of the individualized program based on the OTIPM.

The primary outcome of the study was COPM performance and satisfaction. The COPM was used as a subjective measure of occupational performance. In this evaluation, clients are asked in a semi-structured interview about tasks they want to do, need to do, or are expected to do. For each identified task, performance and satisfaction are self-rated by the clients on a 10-point scale. The COPM was administered in Japanese [11]. The score range was from 1 to 10, and a change of two or more points is considered clinically meaningful [11].

The secondary outcomes of the study were AMPS and ESI. AMPS was used to objectively assess occupation performance and was assessed by the author, an accredited assessor [12]. The AMPS evaluates the degree to which clients perform tasks effortlessly, efficiently, safely, and independently through observation by an occupational therapist. It consists of a total of 36 items: 16 items for motor skills and 20 items for process skills. The score range is from -3 to 4 for motor skills and from -4 to 3 for process skills. The cut-off values are 2.0 for motor skills and 1.0 for process skills [12].

The objective assessment of social interaction skills was conducted by the author, a certified evaluator, using the ESI [13]. The ESI is an observational tool used by occupational therapists to assess whether social interactions are appropriate, mature, polite, respectful, and well-timed. It consists of 27 items, with scores ranging from -2.5 to 2.0, and a cut-off value set at 1.0 [13].

Statistics

COPM, AMPS, and ESI values were compared before and after program implementation. Since the COPM is an ordinal scale, the Wilcoxon signed-rank test was used for pre- and post-intervention comparisons. As AMPS and ESI are interval scales, paired t-tests were used to compare scores before and after the intervention. EZR (Easy R) (Saitama Medical Center, Jichi Medical University, Saitama, Japan) was used for all statistical analyses [14]. The significance level was set at a two-sided risk rate of 5%. G*Power (Ver. 3.1.9.7, Heinrich-Heine-Universität Düsseldorf, Düsseldorf, Germany) was used for power and effect size (Cohen's d) tests. For data with missing values, the median or mean value was substituted, and statistical analysis was performed.

## Results

Subjects' attributes

Participant demographics are presented in Table 3.

**Table 3:** Participant demographics

Category	Detail
Gender	Male: 38, Female: 52
Age (mean)	44.8
Diagnosis	Schizophrenia: 46, Addiction: 15 (Alcohol: 10, Drugs: 5), Mood Disorders: 11 (Bipolar: 6, Depression: 5), Intellectual Disability: 7, Others: 11 (e.g., dissociative disorder, delusional disorder)
Type of hospitalization	Voluntary: 35, Medical Protection: 51, By Measures: 4
Number of admissions (mean/median)	4.9 (median 3.0)
Length of stay until program implementation (mean/median)	528.4 days (median 108.5)
Number of program sessions (mean/median)	6.9 (median 7.0)
The number of clients who received 10 sessions	36

There were 38 male and 52 female subjects with a mean age of 44.8 years. The main illnesses of the subjects were schizophrenia in 46, addiction in 15 (alcoholism in 10, drug addiction in 5), mood disorders in 11 (bipolar disorder in 6, depression/depression in 5), intellectual disability in 7 and others in 11 (dissociative disorder, delusional disorder, etc.). The type of hospitalization was voluntary hospitalization in 35 patients, medical protection hospitalization in 51 patients, and hospitalization for measures in 4 patients. The mean number of admissions was 4.9 (median 3.0), and the mean length of stay until program implementation was 528.4 days (median 108.5 days). The mean number of program runs was 6.9 (median 7.0), with 36 CLs (clients) receiving a maximum of 10 interventions.

Effects before and after the intervention

Table 4 shows the evaluation results before and after the implementation of the individual occupation-based program. A comparison of the whole program before and after is shown in Table 5. COPM performance (p<0.001, Cohen's d=0.79, (1-β)=1.00), satisfaction (p<0.001, Cohen's d=0.97, (1-β)=1.00), AMPS motor skills (p<0.001, Cohen's d=0.61, (1-β)=1.00), process skills (p<0.001, Cohen's d=0.59, (1-β)=1.00), and ESI (p<0.001, Cohen's d=0.72, (1-β)=1.00), with results for all outcome measures. Significant improvements were observed in all outcome measures. At the time of the initial evaluation, the values of the AMPS motor skills, process skills, and ESI were all below the cut-off values, but at the time of the final evaluation, all values had changed to values above the cut-off values.

**Table 4:** Pre- and post-program evaluation results COPM, Canadian Occupational Performance Measure; AMPS: Assessment of Motor and Process Skills; ESI, Evaluation of Social Interaction; Per, performance; Sat, satisfaction; Mot, motor; Pro, process; Pre, pre-program; Post, post-program

Outcome measure	COPM Per (Pre)	COPM Per (Post)	COPM Sat (Pre)	COPM Sat (Post)	AMPS Mot (Pre)	AMPS Mot (Post)	AMPS Pro (Pre)	AMPS Pro (Post)	ESI (Pre)	ESI (Post)
Total (n=90) Average	3.82	5.44	3.80	5.90	1.83	2.08	0.92	1.11	0.81	1.02
Total (n=90) Standard deviation	2.10	2.07	2.95	2.30	0.50	0.44	0.36	0.33	0.34	0.30
Schizophrenia (n=46)	4.40	5.53	4.00	5.90	1.76	2.06	0.84	1.01	0.73	0.96
Addiction (n=15)	3.31	5.42	2.80	6.30	2.02	2.25	1.20	1.29	1.03	1.21
Mood disorder (n=11)	3.33	5.21	3.70	5.90	2.05	2.08	0.99	1.29	0.90	1.15i
Intellectual disability (n=7)	4.00	6.07	2.50	6.60	1.39	1.83	0.79	1.04	0.77	0.87
Other (n=11)	2.47	4.94	3.00	5.90	1.91	2.06	0.88	1.12	0.80	1.03
1-5 times (n=29)	4.00	5.33	3.94	5.71	1.88	2.13	0.84	1.06	0.68	0.94
6-10 times (n=61)	3.74	5.49	3.80	5.90	1.81	2.05	0.95	1.13	0.88	1.06
Within 90 days (n=38)	3.90	5.94	3.75	6.42	2.00	2.15	1.04	1.18	0.91	1.08
91 days or more (n=52)	3.65	4.93	3.80	5.90	1.68	2.00	0.77	0.99	0.73	0.95

**Table 5:** Pre- and post-program comparison **p < 0.01. n = 90 COPM, Canadian Occupational Performance Measure; AMPS: Assessment of Motor and Process Skills; ESI, Evaluation of Social Interaction; Me, median; M, mean; SD, standard deviation; d, Cohen’s d

Outcome measure		Pre	Post	p-value	95% confidence interval	Statistical power	Effect size
COPM performance	Me (Min-Max)	3.68（1.0-10.0）	5.40（1.0-10.0）	p<0.001**	1.19-2.05	1.00	d=0.79
COPM satisfaction	Me (Min-Max)	3.80（1.0-10.0）	5.90（1.0-10.0）	p<0.001**	1.68-2.61	1.00	d=0.97
AMPS motor skills	M±SD	1.83±0.50	2.08±0.44	p<0.001**	0.16-0.33	1.00	d=0.61
AMPS process skills	M±SD	0.92±0.36	1.11±0.33	p<0.001**	0.12-0.25	1.00	d=0.59
ESI	M±SD	0.81±0.34	1.02±0.30	p<0.001**	0.15-0.27	1.00	d=0.72

Furthermore, Table 6 compares the pre- and post-intervention outcomes based on the number of sessions (five or fewer vs. six or more) and the timing of program initiation (within 90 days vs. 91 days or more after admission). In Japan, shortening the length of hospital stays (within 90 days) is strongly encouraged, and therefore, it was considered important to demonstrate the effectiveness of the program even when implemented early or with fewer sessions. Therefore, the participants were divided into two groups based on the length of hospitalization, using 90 days after admission as the cutoff point. Regarding the number of sessions, the cutoff was set at five or fewer versus six or more sessions, as five sessions represented a realistic number that occupational therapists working with clients in the acute phase could feasibly implement in practice. Paired t-tests were used for AMPS and ESI, and the Wilcoxon signed-rank test was used for COPM.

**Table 6:** Pre- and post-program comparisons by program frequency and program start date *p < 0.05, **p < 0.01 COPM, Canadian Occupational Performance Measure; AMPS: Assessment of Motor and Process Skills; ESI, Evaluation of Social Interaction

Outcome measure	1-5 times n=29	6-10 times n=61	Difference between the two groups	Within 90 days n=38	91 days or more n=52	Difference between the two groups
COPM performance	p<0.001**	p<0.001**	p=0.349	p<0.001**	p<0.001**	p=0.195
95% confidence interval	0.5301-2.1292	1.2388-2.2766	-	1.2250-2.8655	0.8621-1.7556	-
Statistical power	0.94	1.00	-	1.00	1.00	-
Effect size	d=0.63	d=0.87	-	d=0.82	d=0.82	-
COPM satisfaction	p=0.001**	p<0.001**	p=0.296	p<0.001**	p<0.001**	p=0.317
95% confidence interval	0.7721-2.77	1.8077-2.8278	-	1.6958-3.3090	1.3189-2.4368	-
Statistical power	0.97	1.00	-	1.00	1.00	-
Effect size	d=0.67	d=1.16	-	d=1.02	d=0.94	-
AMPS motor skills	p<0.001**	p<0.001**	p=0.876	p=0.023*	p<0.001**	p=0.0529
95% confidence interval	0.29-0.788	0.494-0.784	-	0.418-0.803	0.457-0.783	-
Statistical power	0.99	1.00	-	0.76	1.00	-
Effect size	d=0.73	d=0.57	-	d=0.39	d=0.79	-
AMPS process skills	p<0.001**	p<0.001**	p=0.579	p<0.001**	p<0.001**	p=0.229
95% confidence interval	0.367-0.819	0.416-0.743	-	0.295-0.747	0.35-0.728	-
Statistical power	0.96	1.00	-	0.92	1.00	-
Effect size	d=0.65	d=0.56	-	d=0.51	d=0.64	-
ESI	p<0.001**	p<0.001**	p=0.255	p<0.001**	p<0.001**	p=0.335
95% confidence interval	0.0461-0.672	0.554-0.814	-	0.463-0.822	0.278-0.689	-
Statistical power	0.97	1.00	-	0.99	1.00	-
Effect size	d=0.67	d=0.82	-	d=0.63	d=0.79	-

In the comparison based on the number of sessions, significant improvements were observed in all outcome measures in both the group with five or fewer sessions and the group with six or more sessions. In the comparison based on the timing of program initiation, significant improvements were observed in all outcome measures regardless of when the program was started.

Three months after the end of the program, 62 (69%) were discharged from the hospital and 28 (31%) remained in the hospital.

## Discussion

The purpose of this study was to clarify how the implementation of individualized programs based on the OTIPM affects the occupational performance and social interaction skills of clients admitted to a psychiatric hospital. Comparison of the results before and after the implementation of the individualized programs revealed significant improvements in all measures: COPM performance and satisfaction, AMPS motor and process skills, and ESI scores.

In the AMPS, achieving values above the cutoff for motor and process skills is considered an indicator of a person's ability to live independently in the community [12]. Similarly, in the ESI, values above the cutoff suggest a high level of social functioning without major difficulties [13]. Based on these findings, it is reasonable to infer that participation in individualized programs based on OTIPM can enhance clients' occupational performance and social interaction skills.

Hosokawa has noted that evaluating Instrumental Activities of Daily Living (IADL), which involve complex tasks such as using transportation, preparing meals, and performing household chores, can provide insight into an individual's functional status in daily life [15]. IADL is a critical factor for the independent living of clients in the community, and previous research has shown that IADL declines significantly with long-term hospitalization exceeding five years [16]. Therefore, interventions during psychiatric hospitalization targeting IADL are considered essential. In this context, the fact that the participants' AMPS motor and process skills scores exceeded the cutoff is highly meaningful.

The individualized program based on OTIPM was designed to enable clients to engage in meaningful occupations through actual task performance. OTIPM emphasizes a client-centered, occupation-based approach in which therapeutic activities are selected based on the client's individual needs and values.

In the context of instrumental activities of daily living (IADL), this program facilitated direct engagement with essential daily tasks. For example, cooking activities allowed clients to participate in the full process of meal preparation. This included planning the menu, purchasing ingredients, and executing the cooking process itself. These components correspond to critical IADL elements such as the use of executive functions and decision-making in menu planning, application of transportation skills and money management in shopping, and the use of sequencing, tool handling, safety management, and time awareness during cooking.

Furthermore, reflection and problem-solving were integrated through the review of task performance, promoting awareness of improvement strategies for future attempts. Through these structured yet flexible interventions, the OTIPM-based program provided clients with opportunities to address real-life challenges in a supportive therapeutic environment, thereby enhancing their capacity for independent living in the community.

For individuals with chronic schizophrenia, factors associated with higher life satisfaction include being female, maintaining good psychosocial functioning, having reliable confidants, good physical health, and living in a supportive community environment [17]. Among these, good psychosocial functioning and the presence of reliable interpersonal relationships are closely related to strong social interaction skills. Thus, enhancing social interaction skills is crucial for living a satisfying life in the community, beyond merely addressing psychiatric symptoms.

Previous studies have reported improvements in social interaction skills through interventions based on OTIPM, suggesting that OTIPM may contribute to the enhancement of these skills [18]. In the present study as well, ESI scores showed a significant improvement beyond the cutoff value, indicating enhanced social interaction skills. This finding supports the importance of this program in promoting the social functioning necessary for successful community living.

While addressing medical conditions remains fundamental in psychiatric treatment, it can be said that an approach focusing on daily life and occupations, as demonstrated in this program, has strengthened the abilities of the clients. The OTIPM-based individualized approach uniquely emphasizes the spontaneous development of interpersonal interactions, while continuously considering with the client the strategies necessary for engaging in tasks that are meaningful to them [7].

Takita emphasizes that to facilitate societal reintegration during psychiatric hospitalization, it is essential not to rely on uniform assistance methods but to support the expansion of an individual's values and thoughts about life [19]. Providing a variety of life experiences and activities during hospitalization that enable personal agency is crucial, and strengthening the individualized approach is fundamental to achieving this.

Individualized programs based on OTIPM maintain a consistent focus on tasks that are important to the client and reflect each client’s unique values [20]. Simply delivering programs on an individual basis risks becoming a uniform approach (e.g., offering the same brain training exercises to all clients), but OTIPM-based individualized programs, being client-centered and task-centered, are less likely to fall into this pattern. This characteristic may have given clients the experience of having their own values and personal thoughts respected.

While group programs and ward activities often emphasize group conformity, making it difficult to broaden individual experiences, individualized programs provide opportunities for clients to engage in a variety of tasks beyond what is usually available. From this perspective, OTIPM-based individualized programs may offer new possibilities in psychiatric care.

Furthermore, it is noteworthy that the program was effective for both newly admitted and long-term hospitalized clients, even with a small number of sessions. This finding suggests that individualized programs based on OTIPM are effective across both the acute phase (short-term involvement) and the chronic phase (where symptoms are relatively stable). The potential to achieve outcomes within a short intervention period is particularly valuable for psychiatric OTs, who often manage multiple demands, and aligns with the trend toward shorter hospital stays in psychiatric care.

Itami & Kawakami have reported that practice models like OTIPM provide clear direction for occupational therapy and are especially helpful for novice OTs to maintain a focus on occupation in their practice [21]. Similarly, Sirkka et al. reported that using OTIPM helps occupational therapists establish their professional roles and supports continuous quality improvement [22]. In practice settings where OTs have diverse educational backgrounds and years of experience, employing a structured model such as OTIPM is beneficial for ensuring consistent, occupation-centered intervention, regardless of the individual OT responsible. If more OTs were able to incorporate practice models like OTIPM, it could support more clients in maintaining their health and improve the overall quality of occupational therapy practice. Thus, OTIPM may represent a promising new option for psychiatric care.

Future directions and limitations

This study demonstrated that individualized programs based on the OTIPM can lead to improvements in both occupational performance and social interaction skills among psychiatric inpatients. Notably, these effects were observed regardless of the number of sessions or the timing of program initiation. The program's client-centered, occupation-based approach may have enabled more meaningful engagement compared to conventional group-based therapies. The significant improvements in AMPS and ESI scores beyond the established cutoff values support the clinical relevance of the observed changes.

Future studies should consider conducting comparisons between clients receiving the minimal number of sessions (e.g., one session) and those receiving the maximum (e.g., ten sessions) to clarify dose-response relationships. Additionally, further research incorporating follow-up evaluations post-discharge could provide insight into the long-term sustainability of improvements in occupational performance and social interaction skills. It is also recommended that future investigations adopt a multicenter or randomized controlled trial (RCT) design to enhance generalizability and causal inference.

This study has several limitations. First, it did not include a comparison between clients who received only one session and those who received the full set of ten sessions. Such a comparison could have provided a more nuanced understanding of the intervention’s effectiveness depending on intensity. Second, the absence of a control group makes it impossible to definitively attribute the observed improvements solely to the individualized program. Other factors, such as standard occupational therapy interventions or concurrent psychiatric treatments, may have contributed to the outcomes.

## Conclusions

This study found that implementing an individualized program based on the OTIPM for clients admitted to a psychiatric hospital may lead to improvements in both occupational performance and social interaction skills.

Furthermore, the OTIPM-based individualized program is a client-centered approach that aligns with each individual’s values, which is an experience that is often not achievable through conventional programs or standard ward-based care. This uniqueness, combined with its effectiveness regardless of the number of sessions or length of hospitalization, suggests its significant value for future psychiatric care. OTIPM-based individualized programs may serve as a valuable guide for the future direction of occupational therapy in psychiatric settings.
